# Methadone vs Buprenorphine-Naloxone in Opioid Overdose Survivors

**DOI:** 10.1001/jamanetworkopen.2026.29313

**Published:** 2026-08-25

**Authors:** Robert A. Kleinman, Sarah Larney, Naheemot Olaoluwa Sule, Clement Ma, Tanya S. Hauck, Paul Kurdyak

**Affiliations:** 1 Campbell Family Mental Health Research Institute, Centre for Addiction and Mental Health, Toronto, Ontario, Canada; 2 Department of Psychiatry, Temerty Faculty of Medicine, University of Toronto, Toronto, Ontario, Canada; 3 Burnet Institute, Melbourne, Victoria, Australia; 4 ICES, Toronto, Ontario, Canada; 5 Division of Biostatistics, Dalla Lana School of Public Health, University of Toronto, Toronto, Ontario, Canada; 6 Institute of Health Policy, Management and Evaluation, University of Toronto, Toronto, Ontario, Canada

## Abstract

**Importance:**

Individuals who experience opioid overdoses are at high risk of death, and data are limited about the comparative effectiveness of medications for this high-risk population.

**Objective:**

To compare the effectiveness of methadone and buprenorphine-naloxone in individuals with a past-year history of opioid overdose.

**Design, Setting, and Participants:**

This retrospective cohort study with target trial emulation included individuals who started methadone or buprenorphine-naloxone treatment between January 1, 2017, and December 31, 2023, in Ontario, Canada. Participants had a past-year emergency department visit for an opioid overdose and no use of either medication for 7 days. Individuals starting each medication were matched on a 1:1 basis using propensity scores, sex, and index date. Data were analyzed from November 2024 to June 2026.

**Main outcomes and measures:**

The primary outcome was death from any cause within 1 year, analyzed using an initiator approach with Cox proportional hazards regression. The secondary outcomes were opioid overdose and treatment discontinuation. Per-protocol analyses evaluating outcomes before medication discontinuation were also conducted.

**Results:**

A total of 5882 individuals were included in the matched cohort (mean [SD] age, 35.8 [10.8] years; 1888 female [32.1%]). In the methadone-initiating group, 165 individuals (5.6%) died within 1 year, compared with 210 (7.1%) in the buprenorphine-naloxone–initiating group (HR, 0.78; 95% CI, 0.63-0.95; *P* = .01), with an E-value of 1.9. Median time to treatment discontinuation was 25 days (IQR, 7-131 days) in the methadone group compared with 16 days (IQR, 5-69 days) in the buprenorphine-naloxone group (HR, 0.78; 95% CI, 0.74-0.82; *P* < .001). There was no significant difference in the time to first opioid overdose (HR, 1.07; 95% CI, 0.97-1.18; *P* = .17). In the per-protocol analyses, there was no significant difference in the hazard of death among individuals starting methadone and buprenorphine-naloxone (HR, 0.84; 95% CI, 0.48-1.47; *P* = .55) and an increased hazard of opioid overdose among individuals starting methadone (HR, 1.52; 95% CI, 1.19-1.93; *P* < .001).

**Conclusions and Relevance:**

In this matched cohort of individuals with a past-year opioid overdose, starting methadone was associated with a reduced risk of death during the subsequent year compared with starting buprenorphine-naloxone. Treatment durations were short, particularly among individuals starting buprenorphine-naloxone, and there was no significant difference in mortality during receipt of opioid agonist treatment. These findings may be explained by unmeasured confounding, and epidemiologic studies in other jurisdictions are needed to confirm these findings.

## Key Points

**Question:**

Among individuals with a past-year opioid overdose starting opioid agonist treatments, what is the comparative effectiveness of methadone and buprenorphine-naloxone for reducing 1-year mortality?

**Findings:**

In this cohort study of 5882 residents from Ontario, Canada, individuals with a past-year opioid overdose starting methadone had a significantly lower hazard of death over 1 year compared with matched individuals starting buprenorphine-naloxone.

**Meaning:**

In this study, starting methadone after an opioid overdose was associated with a reduced risk of death compared with starting buprenorphine-naloxone, a finding that requires further investigation in other jurisdictions.

## Teaser

This cohort study uses target trial emulation to compare the mortality outcomes associated with methadone vs buprenorphine-naloxone treatment among individuals with a past-year opioid overdose in Canada.

## Introduction

Individuals who experience a nonfatal opioid overdose are at high risk of subsequent death, with estimates of 1-year mortality of 8.6%.^1^ Opioid agonist treatments (OATs), such as methadone and buprenorphine, are the mainstay of treatment for opioid use disorder (OUD), and their use is associated with reductions in all-cause and overdose-specific mortality.^2,3,4,5^ However, there are currently limited data about the comparative effectiveness of OATs in the high-risk subgroup of individuals with recent opioid overdoses in the fentanyl era.^2,3,4^

Retrospective cohort studies have found that starting either methadone or buprenorphine after an opioid overdose is associated with a decreased risk of death; however, these studies have not compared medication effectiveness.^6,7^ Randomized clinical trials (RCTs) comparing OATs often have not described the history of opioid overdose among participants.^8,9^ We conducted a retrospective cohort study using the target trial framework to compare the effectiveness of methadone and buprenorphine-naloxone in reducing mortality among individuals who experienced a past-year opioid overdose during the fentanyl era.

## Methods

### Study Design

We conducted a population-based retrospective cohort study emulating a target trial.^10^ In the target trial framework, a pragmatic RCT is specified and subsequently emulated using observational data. Estimates of effect size and direction in target trial emulations have been found to approximate those of the target trials.^11^ The target trial for this study is specified in eTable 1 in Supplement 1. The target trial would recruit individuals with a history of an emergency department (ED) visit for a nonfatal opioid-involved overdose within the past year who are initiating OAT after at least 7 days without OAT receipt (or 60 days since the last dispensing of extended-release [XR] buprenorphine). Individuals would be randomized to receive either methadone or buprenorphine-naloxone, with dose titration at the discretion of the treating physician. The primary outcome would be all-cause mortality, analyzed in an intention-to-treat manner (primary analysis) and per-protocol manner (secondary analysis). This study was approved by the Centre for Addiction and Mental Health Research Ethics Board and was conducted in accordance with ICES (formerly known as the Institute for Clinical and Evaluative Sciences) policies. The need for informed consent was waived under the Tri-Council Policy Statement 2. The study followed the Reporting of Studies Conducted Using Observational Routinely Collected Health Data Statement for Pharmacoepidemiology (RECORD-PE) and the Reporting of Studies Conducted Using Observational Routinely Collected Health Data (RECORD) extensions of the Strengthening the Reporting of Observational Studies in Epidemiology (STROBE) reporting guidelines.

### Target Trial Emulation

#### Data Sources

We obtained data for the study from ICES, an independent, nonprofit research institute whose legal status under Ontario’s health information privacy law allows it to collect and analyze health care and demographic data, without consent, for health system evaluation and improvement. We obtained sociodemographic and mortality data from the Registered Persons Database; comorbidity and health service utilization data from the Discharge Abstract Database, the Same Day Surgery Database, the National Ambulatory Care Reporting System (NACRS), the Ontario Mental Health Reporting System, and the Ontario Health Insurance Program (OHIP) Claims Database; controlled medication dispensing information from the Narcotic Monitoring System; noncontrolled medication dispensing information from the Ontario Drug Benefit database; and information related to fatal opioid overdoses from the Drug and Drug/Alcohol Related Death (DDARD) database.^12^ These datasets were linked using unique encoded identifiers and analyzed at ICES. All individuals with OHIP coverage are assigned unique encoded identifiers, and linkage is deterministic.

#### Cohort Definition

Ontario residents were included in the cohort if they were aged 15 years or older, had a new initiation of buprenorphine-naloxone or methadone between January 1, 2017, and December 31, 2023, and had a past-year ED visit for an opioid overdose. The accrual window corresponds to a period of increasing and high fentanyl presence in Ontario.^13^ A new initiation was defined as an initiation of methadone or buprenorphine-naloxone occurring after 7 days without medication dispensing or availability of previously dispensed doses, with an adjustment for inpatient admissions.^14^ Individuals were excluded for data quality reasons (death date before the index date), not being an Ontario resident, not being eligible for the OHIP, initiating more than 1 OAT on the index date (including coprescribed slow-release oral morphine), or having an ED visit for an opioid overdose on the index date. We identified OAT dispensing using the Narcotic Monitoring System (eTable 2 in Supplement 1).^14^ If multiple OAT initiations occurred within the year after an ED visit for an opioid overdose, the first treatment initiation was selected. If multiple eligible overdose-treatment pairings occurred during the accrual window, a random visit-initiation pairing was included. ED visits for an opioid overdose were identified using *International Statistical Classification of Diseases and Related Health Problems, Tenth Revision* (*ICD-10*) diagnostic codes T40.0-40.4 and T40.6 in the NACRS, which contains information about all ED visits occurring in Ontario.

#### Covariates

We obtained baseline covariates related to factors with the potential to confound associations between OAT exposure and outcomes. Covariates were selected based on risk factors for the outcomes of mortality, treatment discontinuation, and opioid overdose identified in prior literature, including covariates related to demographics, socioeconomic status, health service utilization, medication use, comorbidities, and past exposure to OAT (eTables 3 and 4 in Supplement 1).^2,14,15,16,17,18,19,20,21,22^ We compared individuals receiving new dispensations of oral methadone with individuals receiving new dispensations of sublingual buprenorphine-naloxone.

#### Assignment Procedures

The randomization process of the target trial was emulated using a matching approach, with a goal of achieving balance of potential confounders between methadone-initiating and buprenorphine-naloxone–initiating groups. Propensity scores were calculated using a logistic regression model from baseline covariates (eTable 4 in Supplement 1). Individuals initiating each medication were then matched in a 1:1 ratio on sex, index date (±90 days), and the logit of the propensity score (with a caliper of 0.2 times the SD of the propensity score).^12^ Matching was conducted using a greedy, nearest-neighbor approach, without replacement. Missing data were assigned a separate category and included in the propensity score calculation. Covariate balance across groups was evaluated using standardized mean differences and comparison of the propensity score distributions between groups.^23^

#### Follow-Up Period and Assignment of Time Zero

We assigned time zero (ie, the index date) to the day of the first medication dispensing that met cohort inclusion criteria. We followed individuals until 1 year after cohort entry, loss of OHIP eligibility, or departure from Ontario.

#### Outcomes

The primary outcome was time to death from any cause. Death was ascertained from the Registered Persons Database, which records date of death for all persons in Ontario, and the DDARD, which contains information from coroner investigations of drug-related deaths in Ontario. The secondary outcomes were time to treatment discontinuation and time to opioid overdose. Treatment discontinuation was defined as 5 consecutive nonhospitalized days without use of the OAT dispensed on the index date. Treatment discontinuation was ascertained from outpatient dispensing records obtained through the Narcotic Monitoring System, with an adjustment for hospitalized days using the Discharge Abstract Database and the Ontario Mental Health Reporting System.^14^ An opioid overdose was defined as either a nonfatal opioid overdose resulting in an ED visit or a fatal opioid overdose. Nonfatal opioid overdoses were ascertained from the NACRS using records of ED visits in Ontario and *ICD-10* diagnostic codes. Fatal opioid overdoses were ascertained from the DDARD.

### Power Calculation

We conducted an a priori power calculation for matched survival outcomes.^24^ A sample size of 1800 matched pairs had greater than 95% power (α = .05) to detect (1) an absolute risk difference of 1.5% in incidence of death between 6.0% and 4.5% over 12 months (hazard ratio [HR], 1.33), or (2) an absolute risk difference of 2.0% in incidence between 10.0% and 8.0% over 12 months (HR, 1.25).

### Statistical Analysis

Data were analyzed from November 2024 to June 2026. We used Cox proportional hazards regression to model time to death. For the secondary outcomes of time to treatment discontinuation and time to opioid overdose, we used cause-specific Cox proportional hazards regression, with competing events of death from any cause and death from another cause, respectively. Given the matched structure of the data, treatment type was the only independent variable within models, and robust variance estimators were used in all models.^25^ We generated cumulative incidence functions for each outcome. In the secondary per-protocol analysis, we evaluated time to death and time to opioid overdose during periods in which individuals were receiving the index course of methadone and buprenorphine-naloxone. For this analysis, individuals were censored either at 5 days without the index OAT (ie, if a discontinuation event occurred) or on the day they were dispensed OATs other than the index OAT (including buprenorphine-XR). We conducted a preplanned sex-based analysis, stratifying the analyses by patient sex assigned at birth. We also conducted a post hoc subgroup analysis, restricting matched pairs of individuals to those who had no OAT use in the previous 3 years. For all models, loss of OHIP eligibility or Ontario residency was treated as a censoring event. We evaluated the proportional hazards assumptions using inspection of log-negative-log and tests based on Schoenfeld residuals.

We conducted sensitivity analyses in which we calculated E-values and modified the event classification criteria in the per-protocol analysis. In the latter analysis, we reclassified deaths occurring more than 1 day after the last dispensed dose as off treatment. Similarly, a nonfatal opioid overdose that occurred more than 1 day after the last dispensing and was followed by a discontinuation event (with no intervening dispensing days) was also classified as off treatment. All *P* values were 2-sided with the level of statistical significance for the primary outcome in the initiator analysis set at α = .05. The secondary outcome, per-protocol, and subgroup analyses should be considered exploratory. Categorical variables were presented as frequencies and percentages, and continuous variables were reported as means with SDs or medians with IQRs, as appropriate. Analyses were conducted with SAS Enterprise Guide, version 8.3.7.202 (SAS Institute Inc).

## Results

**Figure 1.**
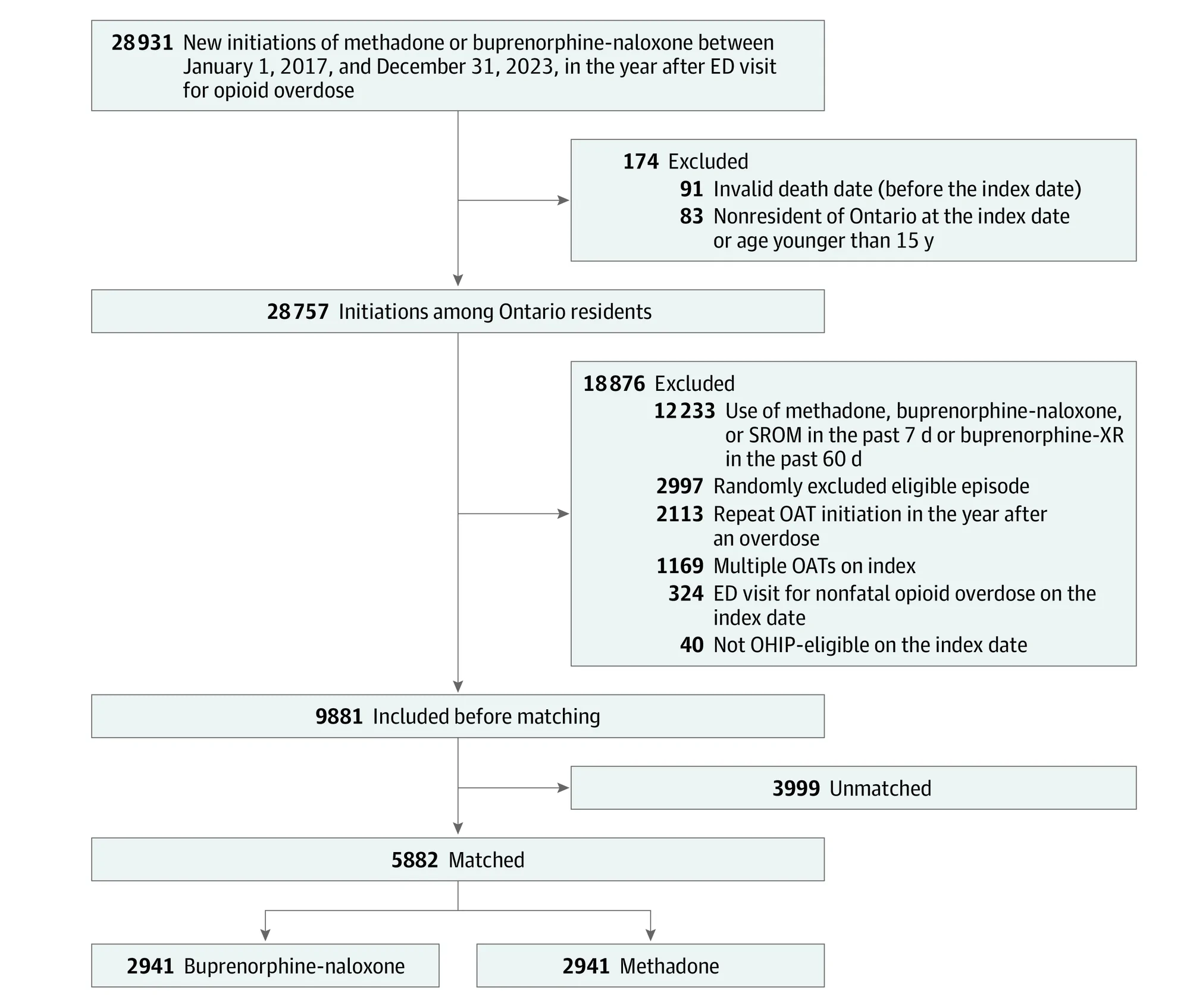
Flowchart of Included and Excluded Individuals ED indicates emergency department; OAT, opioid agonist treatment; OHIP, Ontario Health Insurance Plan; SROM, slow-release oral morphine; XR, extended-release.

A total of 9881 individuals met inclusion and exclusion criteria for the cohort, with 5882 individuals (59.5%) included after matching (mean [SD] age, 35.8 [10.8] years; 1888 female [32.1%] and 3994 male [67.9%]) (Table 1 and Figure 1). Matched individuals had similar characteristics to the overall cohort across most covariates except past OAT exposures. Among matched individuals, 3331 (56.6%) had received OAT in the past 3 years and 2629 (44.7%) had received methadone. Among 3999 unmatched individuals, 3277 (81.9%) had received OAT in the past 3 years, and 2453 (61.3%) had received methadone. Prior methadone exposure was a key covariate that differed between unmatched individuals starting buprenorphine-naloxone and methadone: 1396 individuals (94.6%) starting buprenorphine-naloxone had not previously taken methadone, whereas 2373 unmatched individuals (94.1%) starting methadone had previously taken methadone. Within the matched cohort, a high degree of balance was achieved across measured confounders, with all covariates, including past OAT exposures, having standardized mean differences of 0.1 or less (Table 1; eTable 5 and eFigure 1 in Supplement 1).

**Table 1.** Selected Baseline Characteristics Before and After Matching^a^

Characteristic	Before matching, No. (%)^b^			Standardized mean difference	Matched cohort, No. (%)^b^			Standardized mean difference
	Total (N = 9881)	Buprenorphine-naloxone (n = 4417)	Methadone (n = 5464)		Total (N = 5882)	Buprenorphine-naloxone (n = 2941)	Methadone (n = 2941)	
Age, y								
Mean (SD)	36.45 (11.10)	36.24 (11.57)	36.62 (10.70)	0.034	35.79 (10.84)	35.76 (10.94)	35.82 (10.74)	0.006
15-24	1130 (11.4)	600 (13.6)	530 (9.7)	0.121	765 (13.0)	390 (13.3)	375 (12.8)	0.015
25-34	3889 (39.4)	1696 (38.4)	2193 (40.1)	0.036	2339 (39.8)	1169 (39.7)	1170 (39.8)	0.001
35-44	2773 (28.1)	1171 (26.5)	1602 (29.3)	0.063	1645 (28.0)	815 (27.7)	830 (28.2)	0.011
45-54	1256 (12.7)	562 (12.7)	694 (12.7)	0.001	704 (12.0)	351 (11.9)	353 (12.0)	0.002
55-64	687 (7.0)	311 (7.0)	376 (6.9)	0.006	361 (6.1)	182 (6.2)	179 (6.1)	0.004
≥65	146 (1.5)	77 (1.7)	69 (1.3)	0.039	68 (1.2)	34 (1.2)	34 (1.2)	0
Sex								
Female	3369 (34.1)	1455 (32.9)	1914 (35.0)	0.044	1888 (32.1)	944 (32.1)	944 (32.1)	0
Male	6512 (65.9)	2962 (67.1)	3550 (65.0)	0.044	3994 (67.9)	1997 (67.9)	1997 (67.9)	0
Rurality residence								
Nonrural	8619 (87.2)	3833 (86.8)	4786 (87.6)	0.024	5133 (87.3)	2561 (87.1)	2572 (87.5)	0.011
Rural	1028 (10.4)	504 (11.4)	524 (9.6)	0.059	629 (10.7)	321 (10.9)	308 (10.5)	0.014
Missing	234 (2.4)	80 (1.8)	154 (2.8)	0.067	120 (2.0)	59 (2.0)	61 (2.1)	0.005
Time from overdose to index date, d								
1-7	2077 (21.0)	951 (21.5)	1126 (20.6)	0.023	1106 (18.8)	565 (19.2)	541 (18.4)	0.021
8-28	2213 (22.4)	946 (21.4)	1267 (23.2)	0.043	1178 (20.0)	591 (20.1)	587 (20.0)	0.003
29-180	3940 (39.9)	1756 (39.8)	2184 (40.0)	0.004	2479 (42.1)	1229 (41.8)	1250 (42.5)	0.014
181-365	1651 (16.7)	764 (17.3)	887 (16.2)	0.028	1119 (19.0)	556 (18.9)	563 (19.1)	0.006
Past-year receipt of medication								
Antipsychotics	2469 (25.0)	1215 (27.5)	1254 (23.0)	0.105	1472 (25.0)	739 (25.1)	733 (24.9)	0.005
Antidepressants	2583 (26.1)	1280 (29.0)	1303 (23.8)	0.117	1504 (25.6)	764 (26.0)	740 (25.2)	0.019
Gabapentinoids	1248 (12.6)	627 (14.2)	621 (11.4)	0.085	714 (12.1)	365 (12.4)	349 (11.9)	0.017
Benzodiazepines	2633 (26.6)	1412 (32.0)	1221 (22.3)	0.218	1518 (25.8)	760 (25.8)	758 (25.8)	0.002
Stimulants	1072 (10.8)	526 (11.9)	546 (10.0)	0.061	630 (10.7)	303 (10.3)	327 (11.1)	0.026
Cannabinoid	271 (2.7)	144 (3.3)	127 (2.3)	0.057	154 (2.6)	75 (2.6)	79 (2.7)	0.009
Z-drug	39 (0.4)	26 (0.6)	13 (0.2)	0.055	15 (0.3)	7 (0.2)	8 (0.3)	0.007
Opioids	2509 (25.4)	1278 (28.9)	1231 (22.5)	0.147	1488 (25.3)	766 (26.0)	722 (24.5)	0.034
Any medication covered under ODB	8056 (81.5)	3420 (77.4)	4636 (84.8)	0.19	4758 (80.9)	2379 (80.9)	2379 (80.9)	0
Past-year ED visits								
Opioid overdoses								
1	7400 (74.9)	3316 (75.1)	4084 (74.7)	0.008	4376 (74.4)	2189 (74.4)	2187 (74.4)	0.002
≥2	2481 (25.1)	1101 (24.9)	1380 (25.3)	0.008	1506 (25.6)	752 (25.6)	754 (25.6)	0.002
Nonopioid drug overdoses								
0	8971 (90.8)	3935 (89.1)	5036 (92.2)	0.106	5378 (91.4)	2688 (91.4)	2690 (91.5)	0.002
1	726 (7.3)	367 (8.3)	359 (6.6)	0.066	408 (6.9)	205 (7.0)	203 (6.9)	0.003
≥2	184 (1.9)	115 (2.6)	69 (1.3)	0.097	96 (1.6)	48 (1.6)	48 (1.6)	0
Nonoverdose ED visits								
0	2172 (22.0)	856 (19.4)	1316 (24.1)	0.114	1245 (21.2)	622 (21.1)	623 (21.2)	0.001
1	1937 (19.6)	820 (18.6)	1117 (20.4)	0.047	1137 (19.3)	561 (19.1)	576 (19.6)	0.013
≥2	5772 (58.4)	2741 (62.1)	3031 (55.5)	0.134	3500 (59.5)	1758 (59.8)	1742 (59.2)	0.011
Past-year hospitalizations								
Opioid overdoses								
0	8445 (85.5)	3729 (84.4)	4716 (86.3)	0.053	5087 (86.5)	2540 (86.4)	2547 (86.6)	0.007
1	1332 (13.5)	631 (14.3)	701 (12.8)	0.043	735 (12.5)	371 (12.6)	364 (12.4)	0.007
≥2	104 (1.1)	57 (1.3)	47 (0.9)	0.042	60 (1.0)	30 (1.0)	30 (1.0)	0
Nonopioid drug overdoses								
0	9510 (96.2)	4192 (94.9)	5318 (97.3)	0.126	5690 (96.7)	2827 (96.1)	2863 (97.3)	0.069
1	335 (3.4)	197 (4.5)	138 (2.5)	0.106	181 (3.1)	104-108^c^	73-77^c^	<0.1^c^
≥2	36 (0.4)	28 (0.6)	8 (0.1)	0.078	11 (0.2)	6-10^c^	1-5^c^	<0.1^c^
Nonoverdose hospitalizations								
0	7508 (76.0)	3306 (74.8)	4202 (76.9)	0.048	4527 (77.0)	2245 (76.3)	2282 (77.6)	0.03
1	1532 (15.5)	698 (15.8)	834 (15.3)	0.015	883 (15.0)	444 (15.1)	439 (14.9)	0.005
≥2	841 (8.5)	413 (9.4)	428 (7.8)	0.054	472 (8.0)	252 (8.6)	220 (7.5)	0.04
Johns Hopkins ADGs								
1	15 (0.2)	1-5^c^	10-14^c^	<0.1^c^	1-5^c^	1-5^c^	1-5^c^	<0.1^c^
2	83 (0.8)	28-32^c^	51-55^c^	<0.1^c^	46-50^c^	20-24^c^	21-25^c^	<0.1^c^
3	158 (1.6)	47 (1.1)	111 (2.0)	0.078	84 (1.4)	43 (1.5)	41 (1.4)	0.006
4	269 (2.7)	108 (2.4)	161 (2.9)	0.031	174 (3.0)	86 (2.9)	88 (3.0)	0.004
5	380 (3.8)	134 (3.0)	246 (4.5)	0.077	213 (3.6)	104 (3.5)	109 (3.7)	0.009
6	472 (4.8)	208 (4.7)	264 (4.8)	0.006	280 (4.8)	144 (4.9)	136 (4.6)	0.013
≥7	8504 (86.1)	3887 (88.0)	4617 (84.5)	0.102	5080 (86.4)	2539 (86.3)	2541 (86.4)	0.002
OAT use in past 3 y	6608 (66.9)	2527 (57.2)	4081 (74.7)	0.375	3331 (56.6)	1634 (55.6)	1697 (57.7)	0.043
Longest duration of methadone use in past 3 y								
0	4799 (48.6)	3047 (69.0)	1752 (32.1)	0.795	3253 (55.3)	1651 (56.1)	1602 (54.5)	0.034
1-30 d	1072 (10.8)	383 (8.7)	689 (12.6)	0.128	678 (11.5)	334 (11.4)	344 (11.7)	0.011
31-365 d	2818 (28.5)	750 (17.0)	2068 (37.8)	0.481	1470 (25.0)	724 (24.6)	746 (25.4)	0.017
1-2 y	803 (8.1)	176 (4.0)	627 (11.5)	0.283	359 (6.1)	173 (5.9)	186 (6.3)	0.018
2-3 y	389 (3.9)	61 (1.4)	328 (6.0)	0.247	122 (2.1)	59 (2.0)	63 (2.1)	0.01
Longest duration of buprenorphine-naloxone use in past 3 y								
0	6187 (62.6)	2409 (54.5)	3778 (69.1)	0.304	3612 (61.4)	1819 (61.8)	1793 (61.0)	0.018
1-30 d	1668 (16.9)	731 (16.5)	937 (17.1)	0.016	1043 (17.7)	506 (17.2)	537 (18.3)	0.028
31-365 d	1778 (18.0)	1089 (24.7)	689 (12.6)	0.313	1127 (19.2)	567 (19.3)	560 (19.0)	0.006
1-2 y	194 (2.0)	140 (3.2)	54 (1.0)	0.153	93 (1.6)	44-48^c^	45-49^c^	<0.1^c^
2-3 y	54 (0.5)	48 (1.1)	6 (0.1)	0.127	7 (0.1)	1-5^c^	2-6^c^	<0.1^c^

Abbreviations: ADG, aggregated diagnosis group; ED, emergency department; OAT, opioid agonist treatment; ODB, Ontario Drug Benefit.

^a^ Additional baseline characteristics are presented in eTable 5 in Supplement 1.

^b^ Unless otherwise indicated.

^c^ Data are provided as ranges to prevent ascertainment of counts less than 5.

### Primary Outcome: Mortality

**Figure 2.**
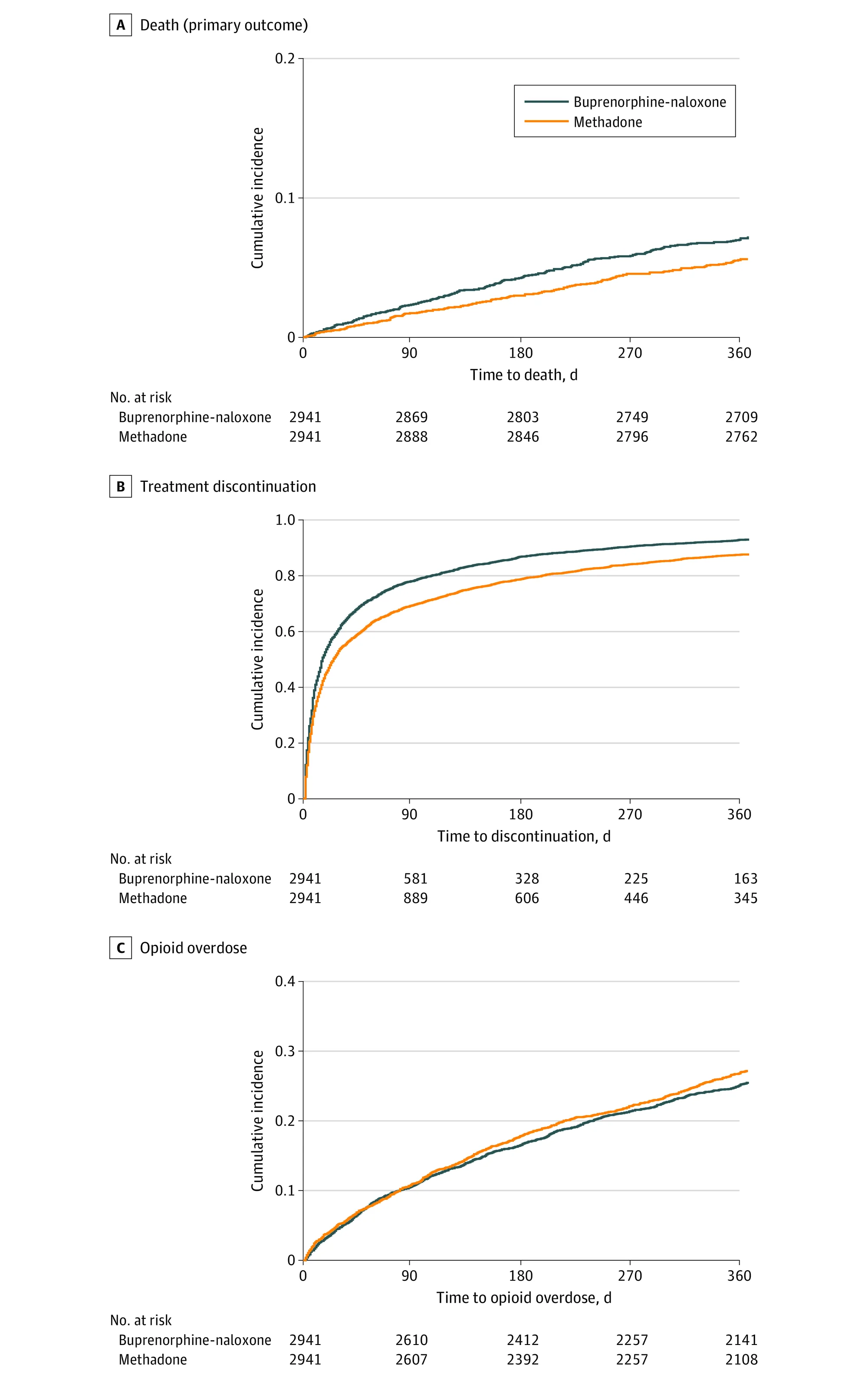
Cumulative Incidence Plots of Outcomes During 1-Year Follow-Up

A total of 375 individuals (6.4%) died within the 1-year follow-up period, including 165 (5.6%) who started methadone and 210 (7.1%) who started buprenorphine-naloxone, with a significantly lower hazard of death in the methadone group (HR, 0.78; 95% CI, 0.63-0.95; *P* = .01) (Table 2 and Figure 2A). Opioid overdose was identified as a cause of death in 264 individuals (4.5% of cohort and 70.4% of decedents). Among individuals who started methadone, 113 (3.8%) experienced a fatal opioid overdose, compared with 151 individuals (5.1%) who started buprenorphine-naloxone. Censoring was less than 1% in both groups (eTable 6 in Supplement 1). The model satisfied the proportional hazards assumption (eFigures 2 and 3 in Supplement 1).

**Table 2.** Results of Primary and Secondary Analyses

Outcome	Initiator (n = 5882)				Per-protocol (n = 5882)			
	No. (%)		Hazard ratio (95% CI)	*P* value	No. (%)		Hazard ratio (95% CI)	*P* value
	Buprenorphine-naloxone	Methadone			Buprenorphine-naloxone	Methadone		
Death	210 (7.1)	165 (5.6)	0.78 (0.63-0.95)	.01	24 (0.8)	26 (0.9)	0.84 (0.48-1.47)	.55
Treatment discontinuation	2605 (88.6)	2577 (87.6)	0.78 (0.74-0.82)	<.001	NA	NA	NA	NA
Opioid overdose	747 (25.4)	797 (27.1)	1.07 (0.97-1.18)	.17	101 (3.4)	180 (6.1)	1.52 (1.19-1.93)	<.001

Abbreviation: NA, not applicable.

### Secondary Outcomes

During the 1-year follow-up, 2577 individuals (87.6%) initiating methadone and 2605 (88.6%) starting buprenorphine-naloxone discontinued the index medication. The median time to treatment discontinuation was 25 days (IQR, 7-131 days) in the methadone group compared with 16 days (IQR, 5-69 days) in the buprenorphine-naloxone group (Figure 2B). There was a lower hazard of discontinuation in the methadone group (HR, 0.78; 95% CI, 0.74-0.82; *P* < .001) (Table 2 and Figure 2B). Censoring was 0.7% (n = 21) among methadone initiators and 5.9% (n = 175) among buprenorphine-naloxone initiators, with 157 individuals (5.3%) censored due to receipt of a buprenorphine-XR (eTable 6 in Supplement 1). Overall, 1544 individuals (26.2%) in the matched cohort experienced an opioid overdose (either fatal or nonfatal) during the 1-year follow-up. In the methadone-initiating group, 797 individuals (27.1%) experienced an opioid overdose, whereas 747 (25.4%) in the buprenorphine-naloxone–initiating group experienced an opioid overdose. There was no significant difference in the time to first opioid overdose (HR, 1.07; 95% CI, 0.97-1.18; *P* = .17) (Figure 2C). The discontinuation model satisfied the proportional hazards assumptions (eFigures 4 and 5 in Supplement 1). For the opioid overdose model, the log-negative-log plot indicated potential nonproportionality, corresponding to differences observed in the per-protocol analyses below (eFigures 6 and 7 in Supplement 1).

### Per-Protocol Analyses

In the per-protocol analyses evaluating outcomes until discontinuation of the index treatment episode, 26 individuals (0.9%) in the methadone group and 24 (0.8%) in the buprenorphine-naloxone group died (Table 2, eTable 7 in Supplement 1). There was no significant difference in the hazard of mortality during treatment (HR, 0.84; 95% CI, 0.48-1.47; *P* = .55). During the period of receipt of the index OAT, there was an increased hazard of opioid overdose while receiving methadone (HR, 1.52; 95% CI, 1.19-1.93; *P* < .001).

### Subgroup Analyses

A sex-stratified subgroup analysis found directionally similar results to those of the primary analysis (Table 3). For the primary outcome of time to death, the HRs comparing individuals starting methadone with those starting buprenorphine-naloxone were 0.70 (95% CI, 0.46-1.05) among females and 0.80 (95% CI, 0.64-1.02) among males. Treatment discontinuation was lower among both females and males starting methadone (females: HR, 0.81; 95% CI, 0.74-0.89; males: HR, 0.77; 95% CI, 0.72-0.82). The per-protocol analysis results were also consistent in females and males (Table 3).

**Table 3.** Sex-Based Subgroup Analysis

Outcome	Females (n = 1888)				Males (n = 3994)			
	No. (%)		Hazard ratio (95% CI)	*P* value	No. (%)		Hazard ratio (95% CI)	*P* value
	Buprenorphine-naloxone	Methadone			Buprenorphine-naloxone	Methadone		
Initiator								
Death	55 (5.8)	39 (4.1)	0.70 (0.46-1.05)	.09	155 (7.8)	126 (6.3)	0.80 (0.64-1.02)	.07
Treatment discontinuation	834 (88.3)	833 (88.2)	0.81 (0.74-0.89)	<.001	1771 (88.7)	1744 (87.3)	0.77 (0.72-0.82)	<.001
Opioid overdose	201 (21.3)	212 (22.5)	1.06 (0.88-1.27)	.56	546 (27.3)	585 (29.3)	1.08 (0.96-1.21)	.21
Per-protocol								
Death	12 (1.3)	7 (0.7)	0.45 (0.18-1.12)	.09	12 (0.6)	19 (1.0)	1.26 (0.61-2.62)	.53
Opioid overdose	24 (2.5)	51 (5.4)	1.80 (1.12-2.88)	.01	77 (3.9)	129 (6.5)	1.43 (1.08-1.90)	.01

An exploratory subgroup analysis among pairs of individuals without any OAT use in the 3 years before the index date (n = 1600) found no differences between methadone-exposed and buprenorphine-naloxone–exposed individuals in hazards of death (HR, 1.00; 95% CI, 0.67-1.50) or opioid overdose (HR, 1.16; 95% CI, 0.94-1.43). Individuals without OAT use in the past 3 years who started methadone were less likely to discontinue treatment (HR, 0.80; 95% CI, 0.72-0.88).

### Sensitivity Analyses

The E-values in the initiator analyses for the point estimates of the time to death and time to treatment discontinuation outcomes were 1.9 and 1.7, respectively. In the sensitivity analysis reclassifying deaths and opioid overdoses that occurred more than 1 day after the last use of OAT as occurring off protocol, results were consistent with the main per-protocol analysis (eTable 8 in Supplement 1). In the 4-day window between day 2 and day 5 after the last dispensed dose, 30 of 2605 individuals (1.2%) discontinuing buprenorphine-naloxone and 22 of 2577 individuals (0.9%) discontinuing methadone experienced an opioid overdose. Seven individuals discontinuing buprenorphine-naloxone (0.3%) and 7 individuals discontinuing methadone (0.3%) died during this period.

## Discussion

This cohort study highlights the high rates of treatment discontinuation, recurrent opioid overdose, and death among individuals starting OAT after a past-year opioid overdose. In this matched cohort, the hazard of death was significantly lower after starting methadone than after starting buprenorphine-naloxone. While individuals were receiving OAT (ie, in the per-protocol analysis), there was no difference in mortality between the methadone and buprenorphine-naloxone groups, and there was a lower hazard of opioid overdose among individuals receiving buprenorphine-naloxone. Treatment duration was short in both groups, though significantly longer among individuals starting methadone.

Our findings of an association between starting methadone after an opioid overdose and lower mortality are consistent with results from a prior retrospective cohort study evaluating mortality outcomes among Massachusetts adults who experienced an opioid overdose between 2012 and 2014, of whom 2040 received methadone and 3022 received buprenorphine.^6^ Although the Massachusetts study was not designed to compare OAT effectiveness, the study estimated adjusted HRs for mortality in months with receipt of each medication compared with months without medication receipt. Adjusted HRs for months with receipt of methadone were 0.47 (95% CI, 0.32-0.71) and with receipt of buprenorphine were 0.63 (95% CI, 0.46-0.87). Our results also extend those from a previous British Columbia study evaluating 2-year mortality outcomes among all individuals starting OAT. In the study, initiating methadone was associated with reduced 2-year mortality among individuals with a history of OAT use and no difference in 2-year mortality among individuals without a history of OAT use.^26^ The decreased hazard of opioid overdose while receiving buprenorphine-naloxone and longer treatment duration with methadone is consistent with prior findings among individuals starting OAT.^12,14,16,26,27^

### Strengths and Limitations

This study has several strengths and makes several important contributions to our understanding of OUD treatment outcomes. First, this study represents the largest cohort of individuals starting OAT after an opioid overdose during the fentanyl era. The short treatment durations, high rates of mortality, and repeat opioid overdose among this cohort of individuals starting recommended OUD treatments highlight the importance of improving treatment outcomes after an opioid overdose. Second, the study provides comparative information about outcomes after OAT initiation in this high-risk population. Our findings suggest that individuals initiating methadone had lower mortality over 1 year than individuals initiating buprenorphine-naloxone, and the new-user, target trial framework lowers, but does not eliminate, risk of bias. These findings require further evaluation through methodologically robust epidemiologic studies and pragmatic trials in other jurisdictions. Third, this study evaluates the comparative effectiveness of methadone and buprenorphine-naloxone in a jurisdiction with similar prescribing frameworks for both medications.^28,29^ Fourth, this study included analyses that showed consistent relative outcomes in males and females and an exploratory subgroup analysis among individuals without OAT use in the past 3 years, suggesting no significant mortality difference within this group.

The study also has several limitations. Although the matched cohort was well-balanced on measured confounders, unmeasured confounding remains possible. The E-value of 1.9 for mortality is of a similar magnitude to other known risk factors for opioid-related mortality, suggesting that study findings could be altered if residual confounding is present.^30,31^ Comorbidities could only be ascertained among health service users. Nonfatal opioid overdoses were only included if they resulted in an ED visit, suggesting rates of opioid overdose within this cohort are higher than ascertained, but this finding would not likely affect comparative outcomes. Because we used an initiator analytic approach to emulate the intention-to-treat framework from RCTs, we did not evaluate time-varying exposures after initiation. The matching rate for eligible individuals from the population-based cohort was 59.5%, potentially limiting the generalizability of findings. In particular, there were differences between matched and unmatched individuals on past OAT exposure. ICES data holdings include information about patient sex but not gender, limiting the potential for gender-based analyses.

## Conclusions

In this cohort study of Ontario residents with a past-year opioid overdose, the hazard of death after starting methadone was significantly lower than after starting buprenorphine-naloxone. Treatment duration was short in both groups but significantly longer among individuals starting methadone, and there were no significant differences in opioid overdoses during the year after OAT initiation. The short treatment durations, high rates of mortality, and repeat opioid overdose among this group of individuals who started recommended treatments for OUD highlight the importance of improving treatment outcomes in this high-risk group. Epidemiologic studies and pragmatic trials in other jurisdictions are needed to confirm these findings and further evaluate outcomes in this high-risk population.

## Notes

Supplement 1. **eTable 1.** Specification of Protocol for the Target Trial **eTable 2.** Drug and Product Identification Numbers Used to Identify Opioid Agonist Treatments **eTable 3.** Definitions of Variables Used in Baseline Characteristics **eTable 4.** Covariates Included in Propensity Score Modelling **eTable 5.** Additional Baseline Characteristics of Included Individuals Before and After Matching **eTable 6.** Reasons for Censoring in Initiator Analyses **eTable 7.** Reasons for Censoring in Per-Protocol Analyses **eTable 8.** Per-Protocol Sensitivity Analysis **eFigure 1.** Density Plots of Propensity Scores Before and After Matching **eFigure 2.** Log of Negative Log Plot for Death Outcome, Initiator Analysis **eFigure 3.** Schoenfeld Residuals for Death Outcome, Initiator Analysis **eFigure 4.** Log of Negative Log Plot for Discontinuation Outcome, Initiator Analysis **eFigure 5.** Schoenfeld Residuals for Discontinuation Outcome, Initiator Analysis **eFigure 6.** Log of Negative Log Plot for Opioid Overdose Outcome, Initiator Analysis **eFigure 7.** Schoenfeld Residuals for Opioid Overdose Outcome, Initiator Analysis

Supplement 2.
Data Sharing Statement
