# Supplementary material for: Methadone vs Buprenorphine-Naloxone in Opioid Overdose Survivors
Source: JAMA Netw Open. 2026 Aug 25;9(8):e2629313. doi: 10.1001/jamanetworkopen.2026.29313 (PMC13507913; doi:10.1001/jamanetworkopen.2026.29313)
Supplement: Supplementary file 1 [file jamanetwopen-e2629313-s001.pdf]

Supplemental Online Content

Kleinman RA, Larney S, Sule NO, et al. Methadone vs buprenorphine-naloxone in opioid overdose survivors. *JAMA Netw Open.* 2026;9(8):e2629313. doi:10.1001/jamanetworkopen.2026.29313.

eTable 1. Specification of Protocol for the Target Trial

eTable 2. Drug and Product Identification Numbers Used to Identify Opioid Agonist Treatments

eTable 3. Definitions of Variables Used in Baseline Characteristics

eTable 4. Covariates Included in Propensity Score Modelling

eTable 5. Additional Baseline Characteristics of Included Individuals Before and After Matching

eTable 6. Reasons for Censoring in Initiator Analyses

eTable 7. Reasons for Censoring in Per-Protocol Analyses

eTable 8. Per-Protocol Sensitivity Analysis

eFigure 1. Density Plots of Propensity Scores Before and After Matching

eFigure 2. Log of Negative Log Plot for Death Outcome, Initiator Analysis

eFigure 3. Schoenfeld Residuals for Death Outcome, Initiator Analysis

eFigure 4. Log of Negative Log Plot for Discontinuation Outcome, Initiator Analysis

eFigure 5. Schoenfeld Residuals for Discontinuation Outcome, Initiator Analysis

eFigure 6. Log of Negative Log Plot for Opioid Overdose Outcome, Initiator Analysis

eFigure 7. Schoenfeld Residuals for Opioid Overdose Outcome, Initiator Analysis

This supplemental material has been provided by the authors to give readers additional information about their work.

© 2026 Kleinman RA et al. *JAMA Network Open*.

eTable 1. Specification of protocol for the target trial

**Protocol Component**	**Description**
Eligibility Criteria	Ontario residents with OUD and an opioid overdose in the past year starting medications for opioid use disorder
Treatment Strategies	Methadone or buprenorphine-naloxone, with dose titration per usual practice
Assignment procedures	Participants will be randomly assigned to a treatment strategy at baseline without blinding
Follow-up period	Starts at randomization and finishes at loss to follow-up or 12 months
Outcomes	Primary: Death; Secondary: Opioid overdose, treatment discontinuation
Causal contrasts of interest	Primary: Intention-to-treat effect; Secondary: Per-protocol effect
Analysis plan	Intent-to-treat and per-protocol analyses of randomized subjects with time-to-event models

© 2026 Kleinman RA et al. *JAMA Network Open*.

eTable 2. Drug and Product Identification Numbers used to identify opioid agonist treatments

Buprenorphine-naloxone	Methadone	Slow-release oral morphine	Buprenorphine-XR	
			Sublocade®	Probuphine®
02408090, 02408104, 02424851, 02424878, 02295695, 02295709, 02468085, 02468093, 02502313, 02502321, 02502348, 02502356, 02453908, 02453916	02481979, 02394596, 02394618, 02495872, 02495880, 02244290, 09850619, 09857499, 02495783	02184435, 02184443, 02184451, 02242163, 02019930, 02019949, 02019957, 02019965, 02177749, 02177757	02483084, 02483092, 09858127, 09858128	02474921

© 2026 Kleinman RA et al. *JAMA Network Open*.

eTable 3. Definitions of variables used in baseline characteristics.

Characteristic	Database	Definition
Age	RPDB	
Sex	RPDB	
Location of residence	RPDB	Recorded location of residence (Ontario Health Region)
Rurality residence	RPDB, PCCF	Rurality was defined using location of residence and the Postal Code Conversion File. A residence was considered rural if the location was in a community with ≤ 10,000 persons
Neighborhood Income Quintile	RPDB, PCCF	Neighborhood income quintile was determined by linking recorded postal codes to Statistics Canada census data and obtaining the income quintile of the associated dissemination area. Quintile 1 reflects the lowest income quintile
Past-year receipt of medication		
Antipsychotics	ODB	Dispensation of medication within specified medication class, within the past year, covered by the Ontario Drug Benefit
Antidepressants	ODB	
Gabapentinoids	ODB	
Any medication covered under ODB	ODB	
Benzodiazepines	NMS	Dispensation of controlled medication within specified medication class, within the past year, recorded within Narcotic Monitoring System
Stimulants	NMS	
Cannabinoid	NMS	
Z-drug	NMS	
Opioids	NMS	
Active prescription on index date		
Benzodiazepines	NMS	Dispensation of controlled medication within specified medication class, on the index date, recorded within Narcotic Monitoring System
Opioids	NMS	
Opioid overdoses		
ED visits in past year	NACRS	ICD-10 Codes: T40.0-40.4, T40.6
Hospitalization in past year	DAD	
Past-year ED visits		
Non-opioid drug overdoses	NACRS	ED visit with ICD-10 codes: X40-X44, X60-X64, X85, Y10-Y14 and exclusion of opioid toxicity codes (T40.0, T40.1, T40.2, T40.3, T40.4, T40.6)
Non-overdose ED visits	NACRS	ED visit with exclusion of ICD-10 codes: X40-X44, X60-X64, X85, Y10-Y14
Mental Health and Addictions	NACRS	ED visit primary ICD-10 codes: F06-F99 or contributory ICD-10 code X60-X84, Y10-Y19, Y28 when primary code is not F06-F99

© 2026 Kleinman RA et al. *JAMA Network Open*.

Past-year hospitalizations		
Non-opioid drug overdoses	DAD	Hospitalization with ICD-10 codes: X40-X44, X60-X64, X85, Y10-Y14 and exclusion of opioid toxicity codes (T40.0, T40.1, T40.2, T40.3, T40.4, T40.6)
Non-overdose hospitalizations	DAD	Hospitalization with exclusion of ICD-10 codes: X40-X44, X60-X64, X85, Y10-Y14
Mental Health and Addictions	DAD, OMHRS	Hospitalization with primary ICD-10 codes: F06-F99 or Contributory ICD-10 code X60-X84, Y10-Y19, Y28 when primary code is not F06-F99<br>OMHRS: Before 2019-20: Any OMHRS record except for primary diagnosis ICD-9 Codes: 290.x, 294.x<br>2019-20 onward: Any OMHRS record except for primary diagnosis ICD-10 Codes: F01.50, F01.51, F02.80, F02.81, F05, G31.84, G31.9, R41.0, R41.9
Mental health comorbidities (past 3 years)		
Mood disorder	NACRS, DAD, OMHRS	Before 2016-17, ICD-10 Codes: F30.x-F34.x, F38.x, F39.x, F53.0<br>After 2016-17, ICD-10 Codes: All above and F06.3<br>OMHRS: Before 2016-17: 296.x, 300.4x, 301.13, 311.x<br>2016-17 to 2018-19: 293.83, 296.x, 300.4x, 301.13, 311.x, 625.4<br>2019-20 onward: F06.3, F31, F32, F33, F34
Anxiety disorders	NACRS, DAD, OMHRS	Before 2016-17, ICD-10 Codes: F40, F41, F42, F43, F48.8, F48.9; F93.1, F93.2<br>After 2016-17, ICD-10 Codes: F06.4, F40, F41, F93.0-2, F94.0<br>OMHRS: Before 2016-17: 300, 300.0x, 300.2x, 300.3x, 308.3x, 309.0x, 309.24, 309.28, 309.3x, 309.4x, 309.8x, 309.9x<br>2016-17 to 2018-19: 293.84, 300, 300.0x, 300.2x, 309.21, 313.23<br>2019-20 onward: F06.4, F40.0x, F40.1x, F40.2x, F41.0x/1x, F41.8x/9x, F93.0, F94.0
Psychotic disorders	NACRS, DAD, OMHRS	Before 2016-17, ICD-10 Codes: F20 (excluding F20.4), F22, F23, F24, F25, F28, F29, F53.1<br>After 2016-17, ICD-10 Codes: All above plus F06.0-2 and F20.4<br>OMHRS: Before 2016-17: 295.x, 297.x, 298.x<br>2016-17 to 2018-19: 293.81/82, 295.x, 297.x, 298.x<br>2019-20 onward: F06.0/1/2, F20.81, F20.9, F22, F23, F25, F28, F29

© 2026 Kleinman RA et al. *JAMA Network Open*.

Substance-related disorders	NACRS, DAD, OMHRS	ICD-10 Codes: F10 to F19, F55<br>OMHRS: Before 2016-17: 291.x (excl. 291.82), 292.x (excl. 292.85), 303.x, 304.x, 305.x<br>2016-17 to 2018-19: 291.x, 292.x, 303.x, 304.x, 305.x<br>2019-20 onward: F10.x-F19.x, Z72.0
Physical comorbidities /complications (past 3 years)		
Hospitalization for endocarditis	DAD	Hospitalization with ICD-10 Codes: I33.0, I33.9, I38, I39, B37.6
Asthma	ASTHMA	Diagnosis based on inclusion within the ICES-derived Ontario asthma cohort. Inclusion within the cohort occurs with ≥1 hospital admission or ≥2 physician billing claims with an asthma diagnosis within two years, occurring at any time prior to index. Associated diagnosis codes: ICD-9: 493; ICD-10: J45, J46; OHIP: 493
Intentional self-harm	NACRS, DAD	ICD-10 Codes: X60-X84, Y10-Y19, Y28 when primary ICD-10 code is not F06-F99
Past-year family physician visits	OHIP Claims	Visit with submission of OHIP billing claim by family physician
Past-year psychiatrist visits	OHIP Claims	Visit with submission of OHIP billing claim by psychiatrist
Medication for opioid use disorder duration/use variables in past 3 years	NMS	Dispensing of medications defined by Drug and Product Identification Numbers in eTable 2
Johns Hopkins ADGs	NACRS, DAD, OHIP Claims, SDS	Johns Hopkins ACG® System Aggregated Diagnosis Groups (ADGs), as categorized by the Johns Hopkins ACG® System Version 10.0.1

RPDB = Registered Persons Database
DAD = Discharge Abstract Database
OHIP = Ontario Health Insurance Program
NACRS = National Ambulatory Care Reporting System
OMHRS = Ontario Mental Health Reporting System
NMS = Narcotic Monitoring System
ODB = Ontario Drug Benefit
PCCF = Postal Code Conversion File
SDS = Same Day Surgery

Class of medication was determined using algorithms developed at ICES based on IQVIA data. Mental health comorbidities are based on ICES algorithms for mental health service utilization.

© 2026 Kleinman RA et al. *JAMA Network Open*.

eTable 4. Covariates included in propensity score modelling.

Variable	Variable Form
Age at index	Categorical
Sex*	Binary
Location of residence (Ontario Health Region)	Categorical
Neighborhood Income Quintile†	Categorical
Eligible for ODB in 1 year lookback	Binary
Receipt of antipsychotics (1 year lookback)	Binary
Receipt of antidepressants (1 year lookback)	Binary
Receipt of gabapentinoid (1 year lookback)	Binary
Receipt of benzodiazepines (1 year lookback)	Binary
Receipt of stimulants (1 year lookback)	Binary
Receipt of cannabinoids (1 year lookback)	Binary
Receipt of opioids, excluding OAT (1 year lookback)	Binary
Active benzodiazepine prescription on index	Binary
Active opioid (excluding OAT) prescription on index	Binary
ED visit for Opioid Overdose in 365 days prior to index	Categorical
ED visit for Other Overdose in 365 days prior to index	Categorical
Non-Overdose ED visits in 365 days prior to index	Categorical
Opioid overdose hospitalization in 365 days prior to index	Categorical
Total Mental health hospitalizations in 365 days prior to index	Categorical
ED visit/Hospitalization for Mood disorders (3 years lookback)	Categorical
ED visit/Hospitalization for Anxiety disorders (3 years lookback)	Categorical
ED visit/Hospitalization for Psychotic disorders (3 years lookback)	Categorical
ED visit/Hospitalization for Intentional self-harm (3 years lookback)	Categorical
Total Family physician/GP visits in 365 days prior to index	Categorical
Total Psychiatrist visits in 365 days prior to index	Categorical
Johns Hopkins ADGs	Categorical
Any OAT use in the past 3 years	Categorical
Any Buprenorphine-XR use in the past 3 years	Categorical
Any SROM use in the past 3 years	Categorical
Longest duration of Methadone in past 3 years	Categorical
Longest duration of Buprenorphine-naloxone in past 3 years	Categorical
Time in days from ED overdose to index date	Categorical

*Exact matching was used for this variable. †Missing data were included as a category. Individuals were only eligible for matching with other individuals with index dates within ± 90 days. Variable categories included in the propensity score calculation are indicated in Table 1 and eTable 5.

eTable 5. Additional baseline characteristics of included individuals before and after matching

	Before matching				After matching			
**Characteristic**	**Total (n=9,881)**	**Buprenorphine-naloxone (n=4,417)**	**Methadone (n=5,464)**	**Standardized Mean Difference**	**Total (n=5,882)**	**Buprenorphine-naloxone (n=2,941)**	**Methadone (n=2,941)**	**Standardized Mean Difference**
Age								
Median (Q1-Q3)	34 (28-43)	34 (28-43)	35 (29-43)	0.055	34 (28-42)	34 (28-42)	34 (28-42)	0.004
Location of residence								
West, n (%)	3,663 (37.1%)	1,613 (36.5%)	2,050 (37.5%)	0.021	2,233 (38.0%)	1,107 (37.6%)	1,126 (38.3%)	0.013
Central, n (%)	1,485 (15.0%)	657 (14.9%)	828 (15.2%)	0.008	903 (15.4%)	448 (15.2%)	455 (15.5%)	0.007
Toronto, n (%)	1,352 (13.7%)	625 (14.1%)	727 (13.3%)	0.025	734 (12.5%)	372 (12.6%)	362 (12.3%)	0.01
East, n (%)	1,996 (20.2%)	884 (20.0%)	1,112 (20.4%)	0.008	1,189 (20.2%)	612 (20.8%)	577 (19.6%)	0.03
North East, n (%)	995 (10.1%)	429 (9.7%)	566 (10.4%)	0.022	593 (10.1%)	289 (9.8%)	304 (10.3%)	0.017
North West, n (%)	390 (3.9%)	209 (4.7%)	181 (3.3%)	0.072	230 (3.9%)	113 (3.8%)	117 (4.0%)	0.007
Neighborhood Income Quintile								
1, n (%)	4,049 (41.0%)	1,685 (38.1%)	2,364 (43.3%)	0.104	2,406 (40.9%)	1,194 (40.6%)	1,212 (41.2%)	0.012
2, n (%)	2,227 (22.5%)	1,013 (22.9%)	1,214 (22.2%)	0.017	1,344 (22.8%)	676 (23.0%)	668 (22.7%)	0.006
3, n (%)	1,436 (14.5%)	630 (14.3%)	806 (14.8%)	0.014	882 (15.0%)	454 (15.4%)	428 (14.6%)	0.025
4, n (%)	1,031 (10.4%)	531 (12.0%)	500 (9.2%)	0.093	597 (10.1%)	291 (9.9%)	306 (10.4%)	0.017
5, n (%)	899 (9.1%)	475 (10.8%)	424 (7.8%)	0.103	531 (9.0%)	265 (9.0%)	266 (9.0%)	0.001
Missing, n (%)	239 (2.4%)	83 (1.9%)	156 (2.9%)	0.064	122 (2.1%)	61 (2.1%)	61 (2.1%)	0
Active prescription on index date								
Benzodiazepines, n (%)	1,237 (12.5%)	687 (15.6%)	550 (10.1%)	0.165	677 (11.5%)	337 (11.5%)	340 (11.6%)	0.003
Opioids, n (%)	678 (6.9%)	360 (8.2%)	318 (5.8%)	0.092	390 (6.6%)	199 (6.8%)	191 (6.5%)	0.011
Past-year family physician visits								
0, n (%)	1,156 (11.7%)	500 (11.3%)	656 (12.0%)	0.021	814 (13.8%)	394 (13.4%)	420 (14.3%)	0.026
1, n (%)	835 (8.5%)	410 (9.3%)	425 (7.8%)	0.054	564 (9.6%)	281 (9.6%)	283 (9.6%)	0.002
2+ visits, n (%)	7,890 (79.9%)	3,507 (79.4%)	4,383 (80.2%)	0.02	4,504 (76.6%)	2,266 (77.0%)	2,238 (76.1%)	0.022
Past-year psychiatrist visits								
0, n (%)	6,749 (68.3%)	2,878 (65.2%)	3,871 (70.8%)	0.122	4,047 (68.8%)	2,025 (68.9%)	2,022 (68.8%)	0.002

© 2026 Kleinman RA et al. *JAMA Network Open*.

1, n (%)	995 (10.1%)	450 (10.2%)	545 (10.0%)	0.007	599 (10.2%)	300 (10.2%)	299 (10.2%)	0.001
2+ visits, n (%)	2,137 (21.6%)	1,089 (24.7%)	1,048 (19.2%)	0.133	1,236 (21.0%)	616 (20.9%)	620 (21.1%)	0.003
Past-year mental health and addictions acute care visits								
ED visits								
0, n (%)	4,764 (48.2%)	1,932 (43.7%)	2,832 (51.8%)	0.162	2,819 (47.9%)	1,380 (46.9%)	1,439 (48.9%)	0.04
1, n (%)	2,741 (27.7%)	1,262 (28.6%)	1,479 (27.1%)	0.034	1,662 (28.3%)	869 (29.5%)	793 (27.0%)	0.057
2+ visits, n (%)	2,376 (24.0%)	1,223 (27.7%)	1,153 (21.1%)	0.154	1,401 (23.8%)	692 (23.5%)	709 (24.1%)	0.014
Hospitalizations, past-year								
0, n (%)	7,925 (80.2%)	3,322 (75.2%)	4,603 (84.2%)	0.226	4,798 (81.6%)	2,394 (81.4%)	2,404 (81.7%)	0.009
1, n (%)	1,346 (13.6%)	710 (16.1%)	636 (11.6%)	0.129	769 (13.1%)	388 (13.2%)	381 (13.0%)	0.007
2+ visits, n (%)	610 (6.2%)	385 (8.7%)	225 (4.1%)	0.188	315 (5.4%)	159 (5.4%)	156 (5.3%)	0.005
Acute care visits for mental health comorbidities (past 3 years)								
Mood disorder								
0, n (%)	8,762 (88.7%)	3,801 (86.1%)	4,961 (90.8%)	0.149	5,220 (88.7%)	2,601 (88.4%)	2,619 (89.1%)	0.019
1, n (%)	756 (7.7%)	411 (9.3%)	345 (6.3%)	0.112	453 (7.7%)	237 (8.1%)	216 (7.3%)	0.027
2+, n (%)	363 (3.7%)	205 (4.6%)	158 (2.9%)	0.092	209 (3.6%)	103 (3.5%)	106 (3.6%)	0.006
Anxiety disorder								
0, n (%)	7,909 (80.0%)	3,391 (76.8%)	4,518 (82.7%)	0.148	4,692 (79.8%)	2,338 (79.5%)	2,354 (80.0%)	0.014
1, n (%)	1,223 (12.4%)	581 (13.2%)	642 (11.7%)	0.043	736 (12.5%)	371 (12.6%)	365 (12.4%)	0.006
2+, n (%)	749 (7.6%)	445 (10.1%)	304 (5.6%)	0.169	454 (7.7%)	232 (7.9%)	222 (7.5%)	0.013
Psychotic disorders								
0, n (%)	9,140 (92.5%)	4,040 (91.5%)	5,100 (93.3%)	0.071	5,430 (92.3%)	2,720 (92.5%)	2,710 (92.1%)	0.013
1, n (%)	405 (4.1%)	203 (4.6%)	202 (3.7%)	0.045	245 (4.2%)	116 (3.9%)	129 (4.4%)	0.022
2+, n (%)	336 (3.4%)	174 (3.9%)	162 (3.0%)	0.053	207 (3.5%)	105 (3.6%)	102 (3.5%)	0.006
Substance-related disorders								
0, n (%)	5,547 (56.1%)	2,369 (53.6%)	3,178 (58.2%)	0.091	3,268 (55.6%)	1,645 (55.9%)	1,623 (55.2%)	0.015
1, n (%)	1,925 (19.5%)	831 (18.8%)	1,094 (20.0%)	0.031	1,157 (19.7%)	559 (19.0%)	598 (20.3%)	0.033
2+, n (%)	2,409 (24.4%)	1,217 (27.6%)	1,192 (21.8%)	0.133	1,457 (24.8%)	737 (25.1%)	720 (24.5%)	0.013
Intentional self-harm								

© 2026 Kleinman RA et al. *JAMA Network Open*.

0, n (%)	6,315 (63.9%)	2,766 (62.6%)	3,549 (65.0%)	0.049	3,782 (64.3%)	1,886 (64.1%)	1,896 (64.5%)	0.007
1, n (%)	2,590 (26.2%)	1,194 (27.0%)	1,396 (25.5%)	0.034	1,528 (26.0%)	765 (26.0%)	763 (25.9%)	0.002
2+, n (%)	976 (9.9%)	457 (10.3%)	519 (9.5%)	0.028	572 (9.7%)	290 (9.9%)	282 (9.6%)	0.009
Physical comorbidities								
Asthma, n (%)	2,785 (28.2%)	1,303 (29.5%)	1,482 (27.1%)	0.053	1,675 (28.5%)	870 (29.6%)	805 (27.4%)	0.049
Endocarditis (past 3 years), n (%)	167 (1.7%)	62 (1.4%)	105 (1.9%)	0.041	89 (1.5%)	43 (1.5%)	46 (1.6%)	0.008
Any buprenorphine-XR receipt (past 3 years), n (%)	124 (1.3%)	70 (1.6%)	54 (1.0%)	0.053	83 (1.4%)	37 (1.3%)	46 (1.6%)	0.026
Any slow-release oral morphine receipt (past 3 years), n (%)	566 (5.7%)	196 (4.4%)	370 (6.8%)	0.102	309 (5.3%)	148 (5.0%)	161 (5.5%)	0.02

© 2026 Kleinman RA et al. *JAMA Network Open*.

eTable 6. Reasons for censoring in initiator analyses

Outcome	Censoring reason	Total (n=5,882)	Buprenorphine-naloxone (n=2,941)	Methadone (n=2,941)
Death (all-cause)	Loss of OHIP, n (%)	45 (0.8%)	28 (1.0%)	17 (0.6%)
	End of follow-up, n (%)	5,462 (92.9%)	2,703 (91.9%)	2,759 (93.8%)
Opioid Overdose	Death, another cause, n (%)	84 (1.4%)	47 (1.6%)	37 (1.3%)
	Loss of OHIP, n (%)	33 (0.6%)	22 (0.7%)	11 (0.4%)
	End of follow-up, n (%)	4,221 (71.8%)	2,125 (72.3%)	2,096 (71.3%)
Discontinuation	Initiation of buprenorphine-XR, n (%)	157 (2.7%)	157 (5.3%)	0 (0.0%)
	Death, n (%)	39 (0.7%)	18 (0.6%)	21 (0.7%)
	End of follow-up or loss of OHIP coverage, n (%)*	504 (8.6%)	161 (5.5%)	343 (11.7%)

*Loss of coverage of OHIP was experienced by 1-5 people total between both exposure groups prior to medication discontinuation.

eTable 7. Reasons for censoring in per-protocol analyses.

Outcome	Censoring reason	Total (n=5,882)	Buprenorphine-naloxone (n=2,941)	Methadone (n=2,941)
Death (all-cause)	Treatment switch, n (%)	535 (9.1%)	286 (9.7%)	249 (8.5%)
	Treatment discontinuation, n (%)	4,818 (81.9%)	2,469 (84.0%)	2,349 (79.9%)
	End of follow-up or loss of OHIP coverage, n (%)*	479 (8.1%)	162 (5.5%)	317 (10.8%)
Opioid Overdose	Treatment switch, n (%)	509 (8.7%)	279 (9.5%)	230 (7.8%)
	Treatment discontinuation, n (%)	4,630 (78.7%)	2,394 (81.4%)	2,236 (76.0%)
	Death, another cause, n (%)	18 (0.3%)	8 (0.3%)	10 (0.3%)
	End of follow-up or loss of OHIP coverage, n (%)*	444 (7.5%)	159 (5.4%)	285 (9.7%)

*Loss of coverage of OHIP was experienced by 1-5 people total between both exposure groups prior to medication discontinuation.

© 2026 Kleinman RA et al. *JAMA Network Open*.

eTable 8. Per-protocol sensitivity analysis. In this analysis deaths and opioid overdoses occurring between 2 and 5 days after the last day of availability of the index OAT are considered off protocol. In the main per-protocol analysis, these events were considered as on protocol given that 5 days were necessary for a discontinuation event to be defined. Hazard ratios compare methadone with the buprenorphine-naloxone reference.

	Buprenorphine-naloxone, n (%)	Methadone, n (%)	Hazard Ratio (95% CI)	*P* value
**Death**	17 (0.6%)	19 (0.6%)	0.85 (0.44-1.65)	.63
**Opioid overdose**	71 (2.4%)	158 (5.4%)	1.83 (1.38-2.42)	<.001

© 2026 Kleinman RA et al. *JAMA Network Open*.

eFigure 1. Density plots of propensity scores before and after matching

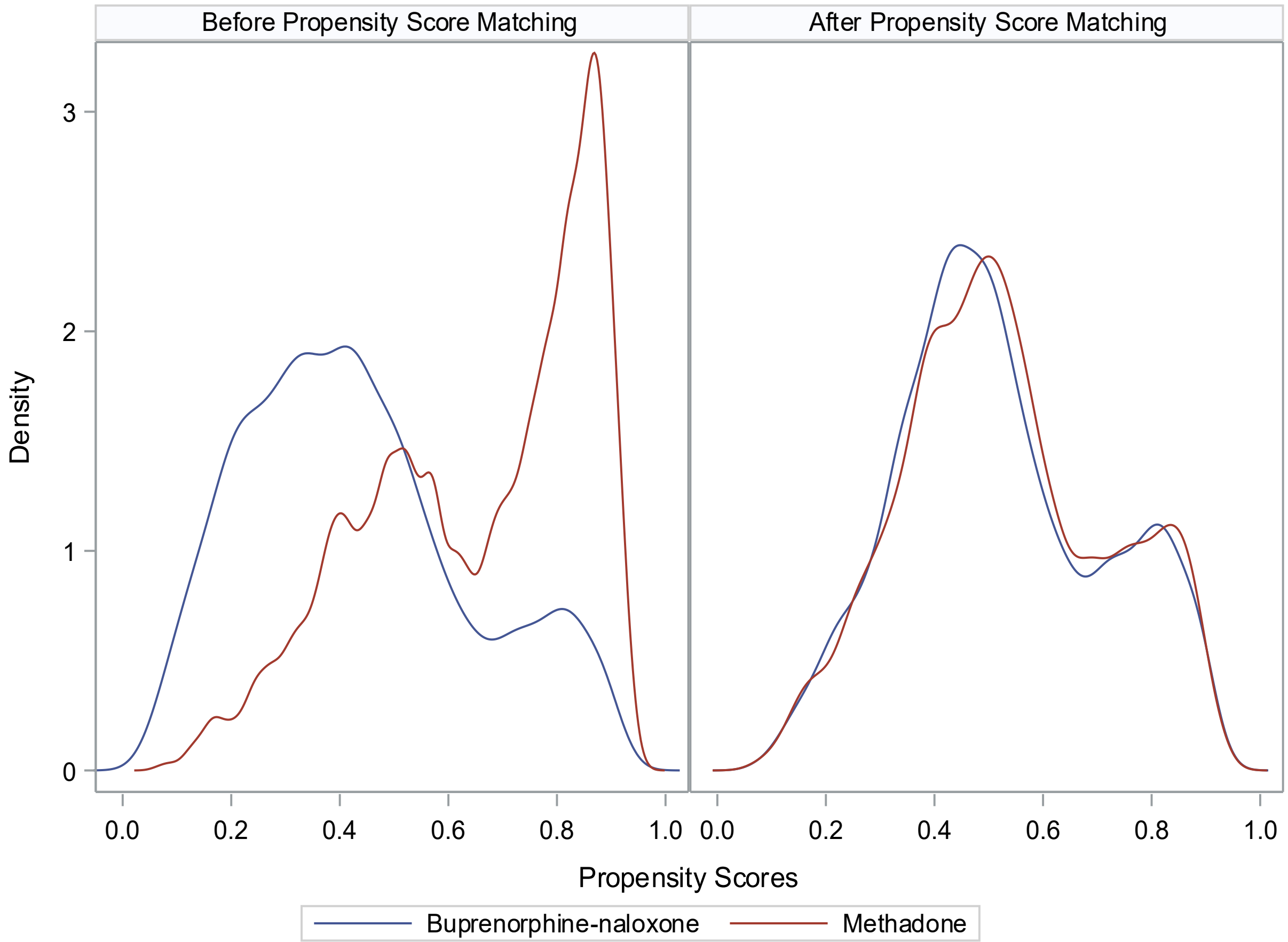

Density plots reflect the distribution of propensity scores among individuals starting buprenorphine-naloxone and methadone before and after matching.

© 2026 Kleinman RA et al. *JAMA Network Open*.

eFigure 2. Log of Negative Log Plot for Death Outcome, Initiator Analysis.

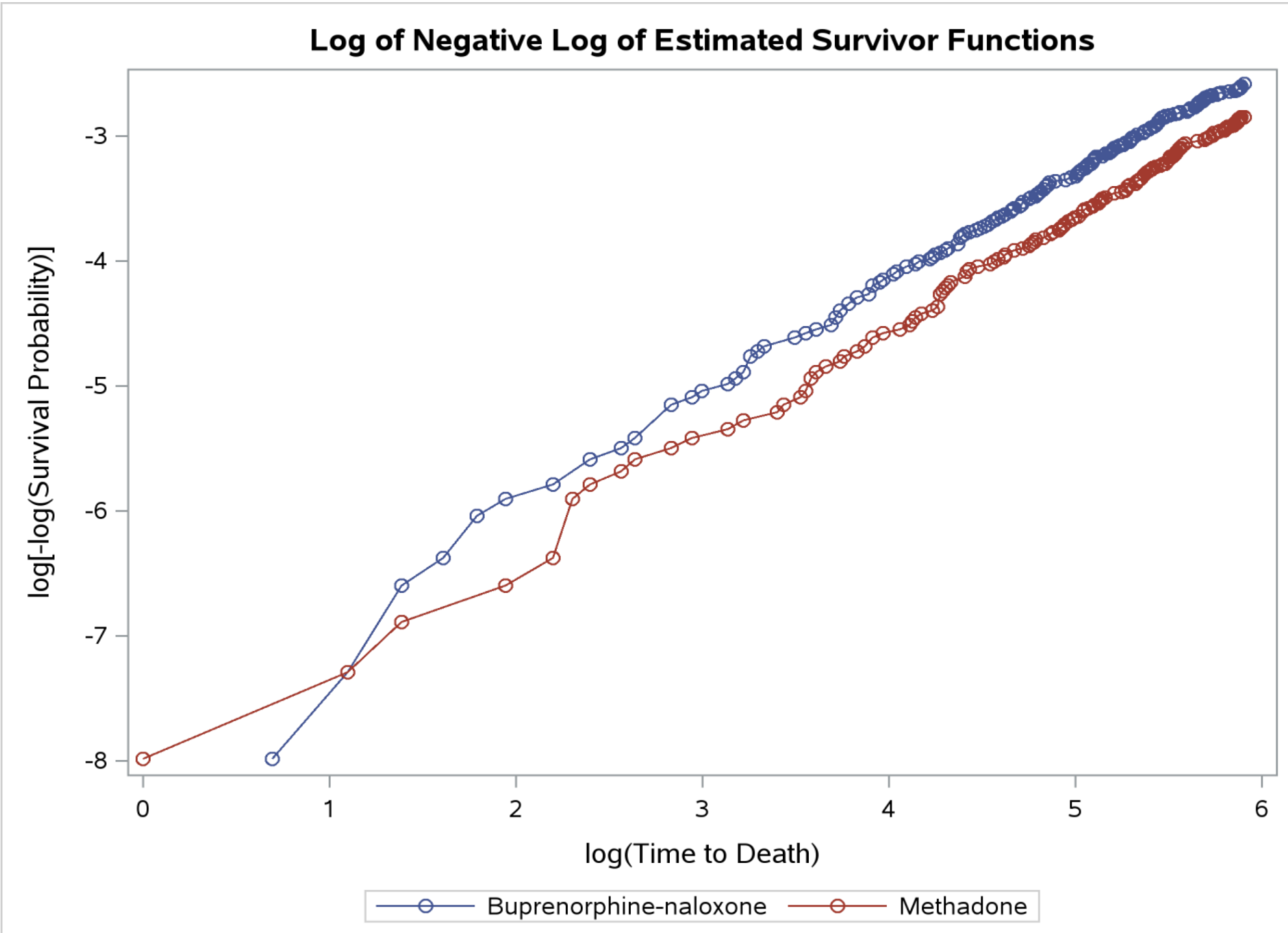

The log of negative log plot for death indicated that the proportional hazards assumption was not violated for the Cox model.

© 2026 Kleinman RA et al. *JAMA Network Open*.

eFigure 3. Schoenfeld residuals for Death Outcome, Initiator Analysis.

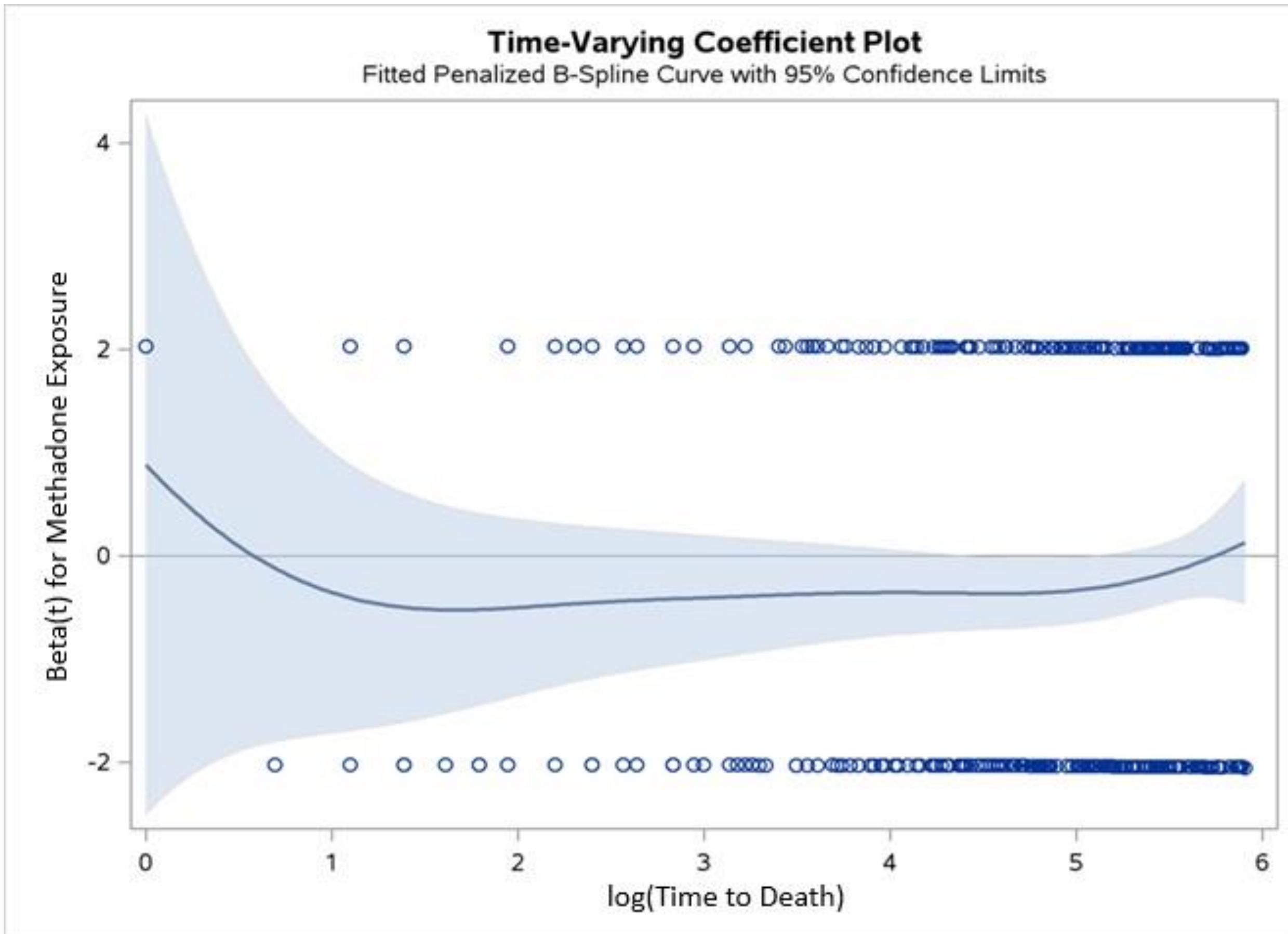

The Schoenfeld residuals plot for death and zph test for non-proportional hazards (p=0.34) indicated that the proportional hazards assumption was not violated for the Cox model.

© 2026 Kleinman RA et al. *JAMA Network Open*.

eFigure 4. Log of Negative Log Plot for Discontinuation Outcome, Initiator Analysis.

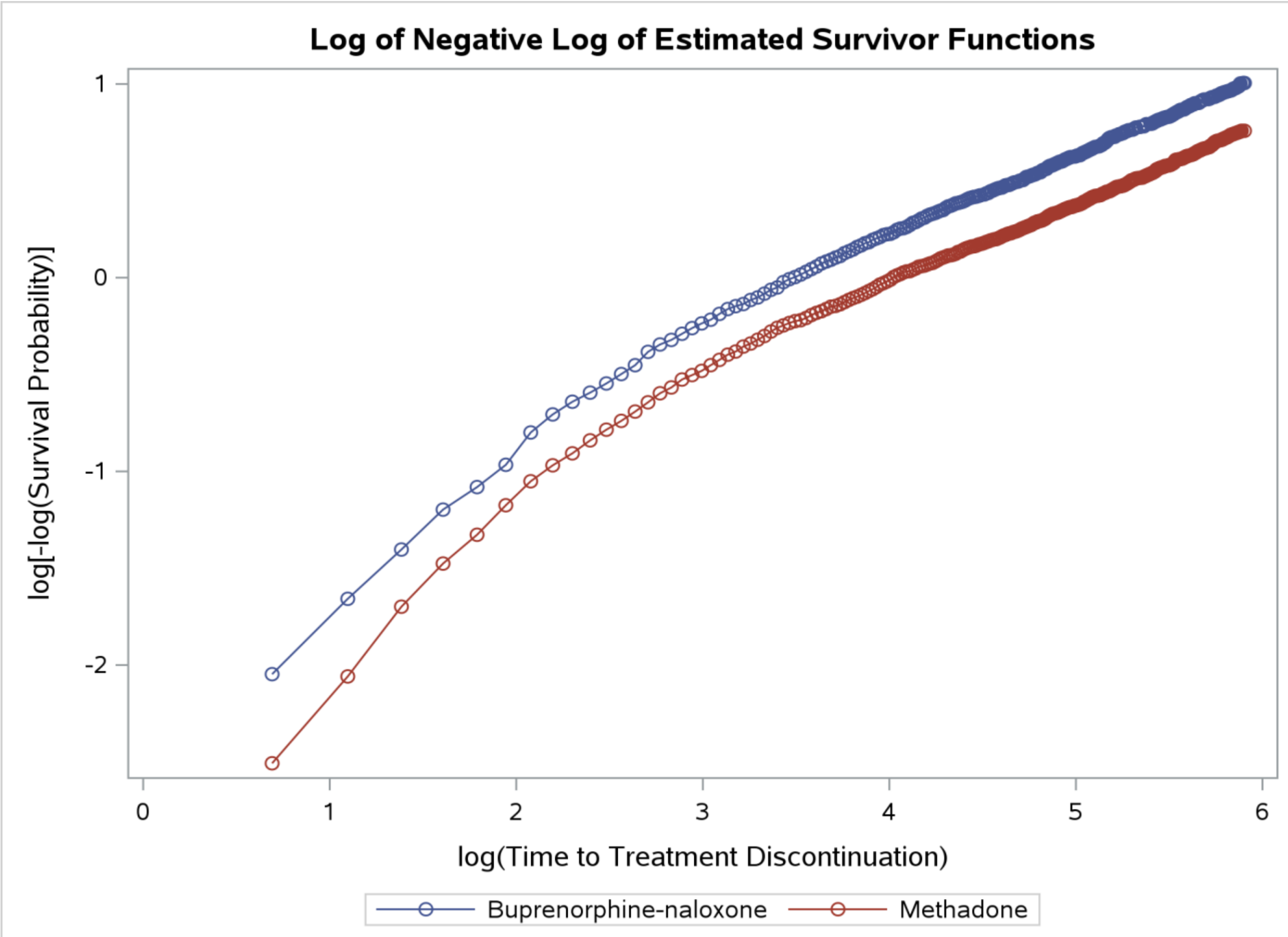

The log of negative log plot for discontinuation indicated that the proportional hazards assumption was not violated for the Cox model.

© 2026 Kleinman RA et al. *JAMA Network Open*.

eFigure 5. Schoenfeld residuals for Discontinuation Outcome, Initiator Analysis.

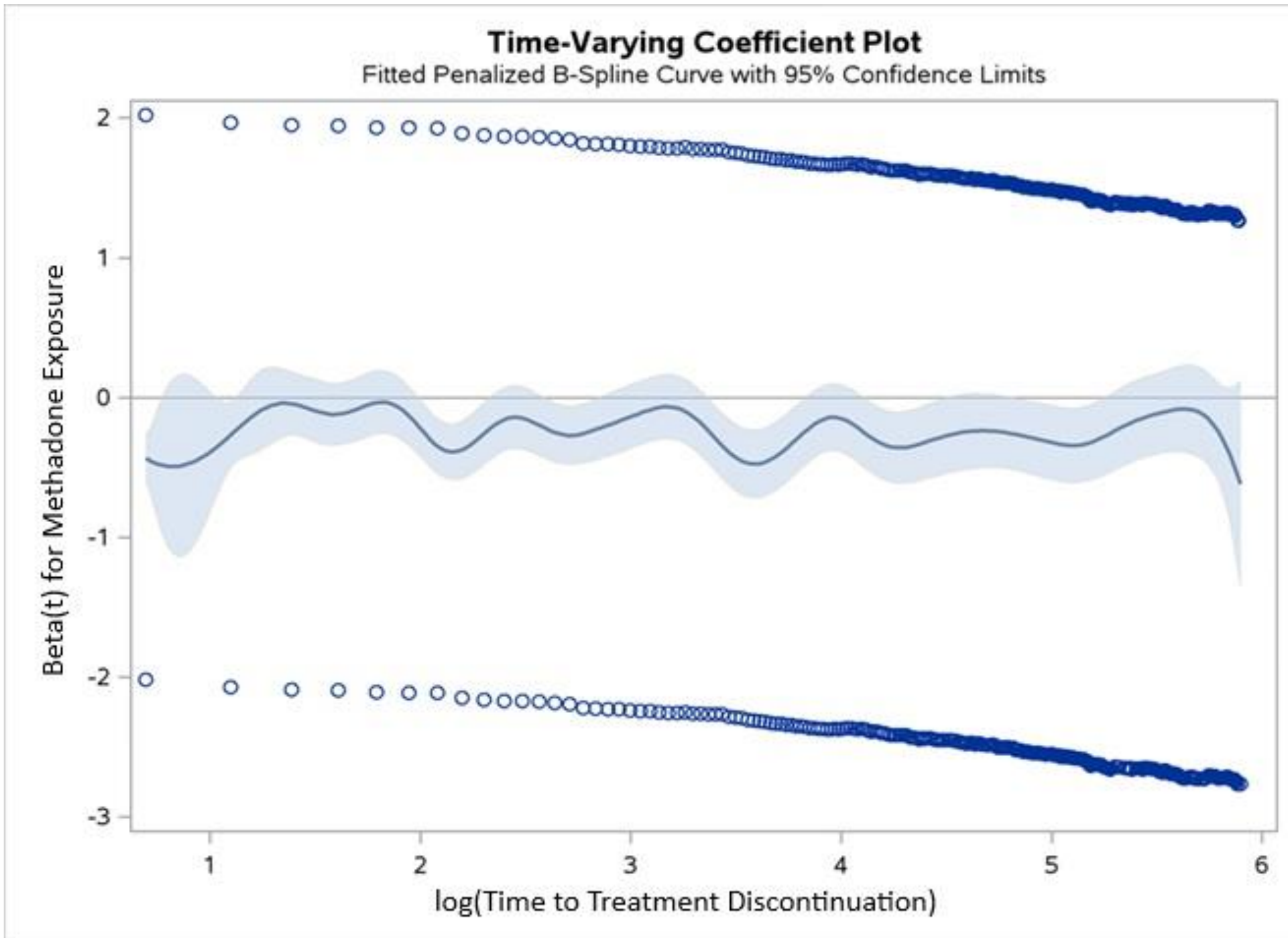

The Schoenfeld residuals plot for discontinuation and zph test for non-proportional hazards (p=0.74) indicated that the proportional hazards assumption was not violated for the Cox model.

© 2026 Kleinman RA et al. *JAMA Network Open*.

eFigure 6. Log of Negative Log Plot for Opioid Overdose Outcome, Initiator Analysis.

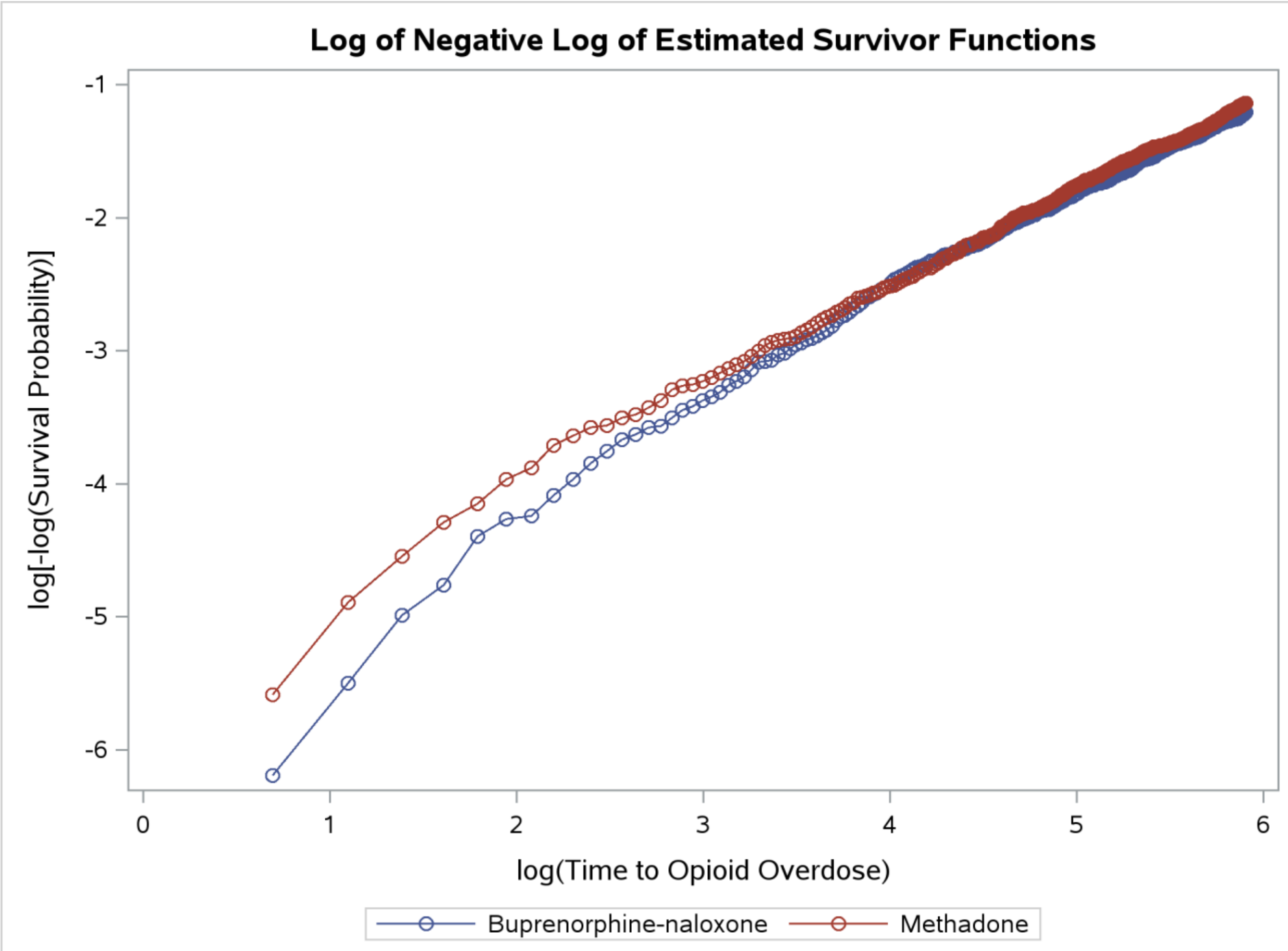

Inspection of the log of negative log plots for opioid overdose revealed possible violation of the proportional hazards assumption. Non-proportionality within this model was addressed through the per-protocol analysis.

© 2026 Kleinman RA et al. *JAMA Network Open*.

eFigure 7. Schoenfeld residuals for Opioid Overdose Outcome, Initiator Analysis.

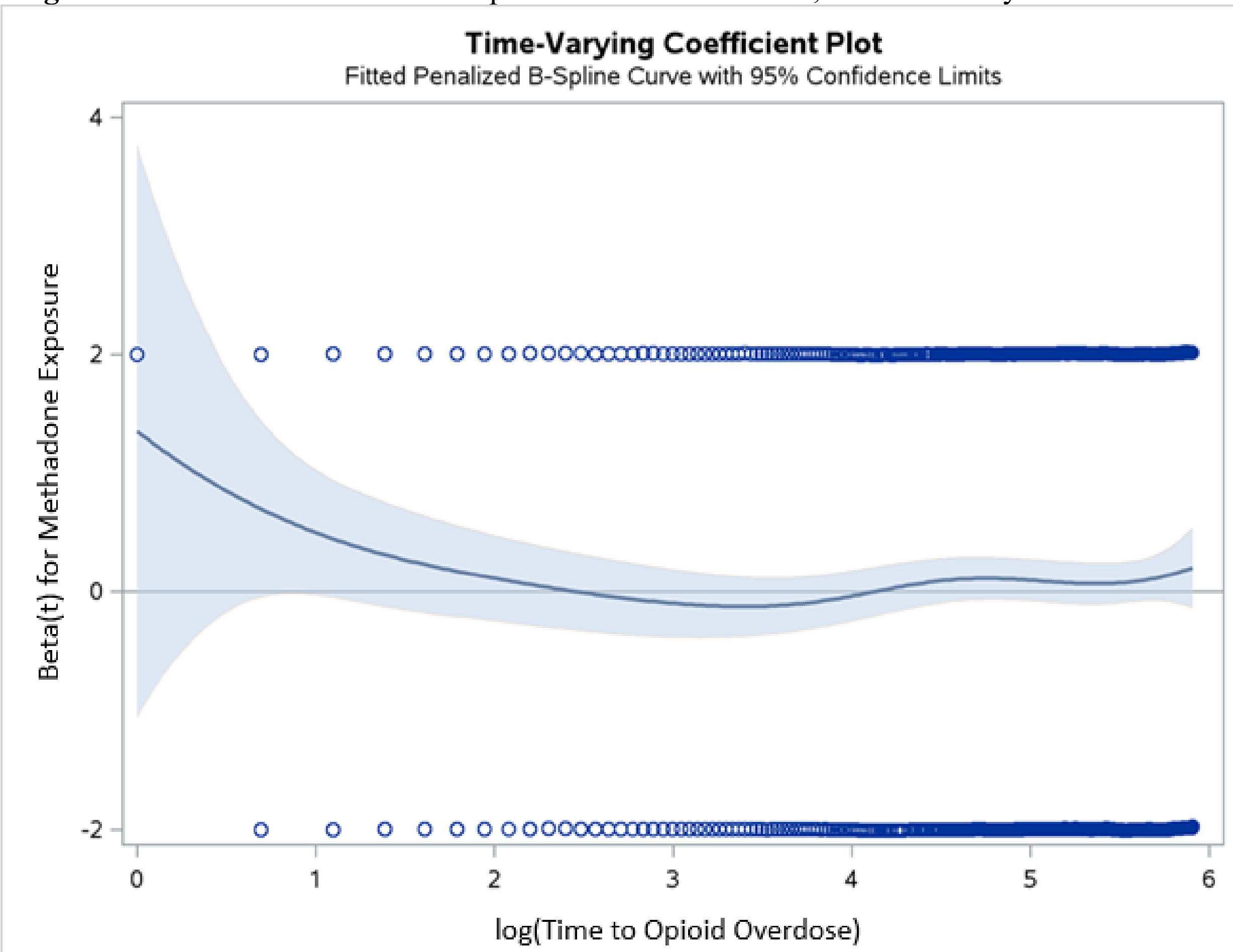

The Schoenfeld residuals plot for opioid overdose and zph test for non-proportional hazards (p=0.99) indicated that the proportional hazards assumption was not violated for the Cox model, though graphical inspection (eFigure 6) revealed a possible violation. Non-proportionality within this model was addressed through the per-protocol analysis.

© 2026 Kleinman RA et al. *JAMA Network Open*.
